# Prevalence of hepatitis B in people living with HIV/AIDS in Latin America and the Caribbean: a systematic review and meta-analysis

**DOI:** 10.1186/s12879-017-2695-z

**Published:** 2017-08-24

**Authors:** Fatima Mitiko Tengan, Edson Abdala, Marisa Nascimento, Wanderley Marques Bernardo, Antonio Alci Barone

**Affiliations:** 1 Department of Infectious and Parasitic Diseases, School of Medicine, University of São Paulo (Universidade de São Paulo - USP), São Paulo, Brazil; 2 Laboratory of Viral Medical Research in Hepatology (Laboratório de Investigação Médica em Hepatologia por vírus - LIM-47), Clinical Hospital, School of Medicine, USP, São Paulo, Brazil; 3 Nursing Division, Clinics Hospital, School of Medicine, USP, São Paulo, SP Brazil; 4 School of Medicine, USP, São Paulo, Brazil; 5 Brazilian Medical Association, Sao Paulo, Brazil

**Keywords:** Hepatitis B, Hepatitis B virus, HIV, Human immunodeficiency virus, Latin America, The Caribbean, Prevalence, Systematic review, Review

## Abstract

**Background:**

Hepatitis B virus (HBV) infection is a major cause of chronic liver disease worldwide. In immunocompromised patients, the chronicity rates of HBV infection are higher, but the rates of hepatitis Be antigen (HBeAg) and HBsAg loss and seroconversion to anti-HBe and anti-HBs are lower than those in immunocompetent subjects. This study aimed to evaluate articles on the prevalence of HBsAg in people living with human immunodeficiency virus (HIV) /AIDS (PLWHA) in Latin America and the Caribbean (LAC).

**Methods:**

We searched the PubMed, Latin American and Caribbean Health Sciences, and Embase databases for studies up to November 2016 on infection with HIV and HBV in LAC without period or language restrictions. We did not include case reports, case series, review articles, comments, or studies with a sample size smaller than 100. We also evaluated the quality of the articles using a list of criteria totaling 21 items.

**Results:**

Of the 28 selected articles (*n* = 18,457) published from 1999 to 2016, 18 studies (64.3%) were from Brazil, 3 (10.7%) were from Argentina, 2 (7.1%) were from Chile, 2 (7.1%) were from Cuba, 1 (3.6%) was from Colombia, 1 (3.6%) was from Venezuela, and 1 (3.6%) was from Jamaica. The mean score for the assessment of the study quality was 11.6 (range: 8–16). The estimated pooled prevalence of HBsAg among PLWHA in the selected studies was 7.0% (95% CI 7.0–7.0%). The pooled prevalence of HBsAg was 8.0% (95% CI 8.0–9.0%) in the studies published from 1999 to 2006 and 6.0% (95% CI 5.0–6.0%) in the studies published during the later timeframe.

**Conclusions:**

The results of this review indicate the need to increase the investment in preventive measures against hepatitis B, particularly when the impact of adequate vaccination in this population is considered. Future studies with larger sample sizes are needed in LAC to determine the true prevalence of hepatitis B throughout the region and to clarify and address the risk factors associated with the acquisition of infection.

**Electronic supplementary material:**

The online version of this article (10.1186/s12879-017-2695-z) contains supplementary material, which is available to authorized users.

## Background

Hepatitis B virus (HBV) infection is a major cause of chronic liver disease worldwide, and approximately 350 million people are chronically infected. In the absence of antiviral therapy, 15% to 40% of patients positive for the hepatitis B surface antigen (HBsAg) develop progressive liver disease, cirrhosis, hepatocellular carcinoma, and terminal liver failure [1]. Because HBV is primarily transmitted via the parenteral and sexual routes and during the perinatal period similar to human immunodeficiency virus (HIV), coinfection with HIV and HBV is common [2].

HIV infection appears to have a negative impact on the natural history of HBV infection. In immunocompromised patients, the chronicity rates of HBV infection are higher, but the rates of hepatitis Be antigen (HBeAg) and HBsAg loss and seroconversion to anti-HBe and anti-HBs are lower than those in immunocompetent subjects [3–5].

Patients with HIV infection have lower serum alanine aminotransferase and albumin levels and higher serum HBV DNA levels than patients with HBV monoinfection. Additionally, the prevalence of cirrhosis is higher in HIV-positive patients [6]. Coinfection with HIV and HBV is associated with a higher rate of HBV reactivation and an increased incidence of cirrhosis and death from cirrhosis in cases with low CD4 counts. Coinfection is also associated with a higher incidence of hepatocellular carcinoma [7, 8].

Other studies have suggested that HBV is associated with a rapidly progressive course of HIV infection [9]. A retrospective analysis indicated that the risk of death in 64 individuals coinfected with HIV and HBV was approximately two-fold higher than that in individuals with HIV monoinfection [10].

The estimated prevalence of hepatitis B among people living with HIV/AIDS (PLWHA) is 5–20%. Therefore, approximately 2 to 4 million people living with HIV have chronic hepatitis B coinfection [2, 11]. Although several studies have evaluated the prevalence of hepatitis B in PLWHA in Latin America and the Caribbean (LAC), the results are conflicting among the evaluated regions and even within the same geographic area. This study aimed to evaluate the studies on the prevalence of HBsAg in LAC countries or territories.

## Methods

We conducted a systematic review of published articles on the prevalence of HBV infection in PLWHA in LAC countries or territories. Our review was conducted and reported in accordance with the Preferred Reporting Items for Systematic Reviews and Meta-Analyses (PRISMA) Statement published in 2009 [12] (Additional file 1).

### Search strategies

We searched Medline via the PubMed, Latin American and Caribbean Health Sciences (Literatura Latino-Americana e do Caribe em Ciências da Saúde–Lilacs) via BVS, and Embase databases for studies up to November 2016 on infection with HIV and HBV in LAC without period and language restrictions. In Medline, we used the terms [(HIV OR Acquired Immunodeficiency Syndrome Virus OR AIDS OR HTLV OR Human Immunodeficiency Virus OR Human TCell) AND (HBV OR Hepatitis B OR Hepatitis B Virus) AND (name of each country or territory from LAC)]. In Lilacs, we used the terms [(HIV OR AIDS OR HUMAN IMMUNODEFICIENCY VIRUS) [Words] AND (HBV OR HEPATITIS B) [Words]]. In Embase, we searched the terms [(HIV OR AIDS OR HUMAN IMMUNODEFICIENCY VIRUS) AND (HBV OR HEPATITIS B)]. We manually searched the references of the selected studies and reviewed articles on the subjects to identify other relevant studies. Disagreements on the identification of relevant studies were discussed until a consensus was reached.

The studies eligible for reading of the titles and abstracts were selected independently by two researchers (E.A. and M.N.), and a list of potentially relevant studies was generated. Articles for inclusion in the review were selected after reading the full text (A.A.B. and W.M.B.).

The articles included were those that contained data on HBV infection in PLWHA, a serological diagnosis of HIV and HBV, and estimates of the prevalence of HBsAg in HIV-infected individuals.

### Study selection

We included original articles that reported the prevalence of HBV in PLWHA in LAC with a sample size ≥100. We did not include case reports, case series, review articles, comments, studies whose participants did not live in LAC, or studies that contained the same case series presented in other publications. Regarding the latter studies, the article with the most complete data was included in the study. We excluded self-reported HIV and/or HBV infections, data obtained from mandatory reporting of HIV and/or HBV (e.g., databases of the local Ministry of Health), and studies whose participant selection method was unclear.

We used the following definitions: (1) HIV infection: the presence of anti-HIV antibodies measured by enzyme immunoassay; (2) HBV infection: the presence of HBsAg; and (3) Latin America and the Caribbean: the countries and territories of Argentina, Bolivia, Brazil, Chile, Colombia, Ecuador, French Guiana, Guyana, Paraguay, Peru, Suriname, Uruguay, Venezuela, Belize, Costa Rica, El Salvador, Guatemala, Honduras, Mexico, Nicaragua, Panama, Aruba, Antigua and Barbuda, Aruba, Bahamas, Barbados, Bonaire, British Virgin Islands, Cayman Islands, Cuba, Curaçao, Dominica, Dominican Republic, Grenada, Guadalupe, Haiti, Jamaica, Martinique, Montserrat, Puerto Rico, Saba, Saint Barthelemy, Saint Kitts and Nevis, Saint Lucia, Saint Martin, Saint Vincent and The Grenadines, Sint Eustatius, Sint Maarten, Trinidad and Tobago, Turks and Caicos Islands, and the United States Virgin Islands.

### Data extraction

Data were collected independently by two investigators (A.A.B. and W.M.B.), and disagreements were resolved via discussions and a consensus. The following data were collected from the articles: author name(s), year of publication, country (state in the case of Brazilian studies), period of data collection, type of study, sample size, mean age, gender of the participants, and number of HBsAg-positive individuals.

Some studies did not report all of the variables necessary for the calculation of prevalence. In these cases, the missing variables were calculated using other reported values ​​(e.g., the numerator was calculated from the values of the denominator and the prevalence).

### Quality assessment of the studies

Based on the criteria proposed by Boyle [13], Fowkes and Fulton [14], Loney [15], and Prins [16], we created a list of criteria related to the **adequacy of the sample** (11 items: adequate design, prospective collection, definition of the target population, probability sampling, sample size calculation, inclusion and exclusion criteria, specification of the collection period, specification of the age range, adequate participant selection, acceptable sample loss, and sample representativeness), **data collection** (4 items: standardization of collection, clear definition of the outcome, definition of the outcome, and description of the outcome measurement), and **data analysis and presentation** (6 items: description of statistical analysis, total number of participants, number of events (outcomes), prevalence by sex and age, prevalence with confidence interval, and satisfactory confidence interval), for a total of 21 items. The items were scored as positive or negative, and the importance of each item was not weighted. Higher scores (positive items) corresponded to higher-quality studies for our review. This assessment was made independently by two researchers (E.A. and M.N.). Disagreements in the evaluation of the quality of the studies were discussed, and a consensus was reached.

### Statistical analysis

As a function of the expected heterogeneity among the selected studies, all meta-analyses were performed using random-effects models based on the DerSimonian and Laird method, which took into account variation among studies. Heterogeneity was assessed using Cochran’s Q statistic (expressed as χ2 and *p* values) and the I2 statistic, which described the percentage of variation among studies explained by heterogeneity rather than by chance. I2 values higher than 25%, 50%, and 75% are considered evidence of low, moderate, and high heterogeneity among studies, respectively. Low I2 values indicate that the variability among estimates is compatible with random variation. Additionally, we investigated potential sources of heterogeneity using a meta-regression analysis. The following factors were assessed using both uni- and multivariate models: publication year (comparison between studies published from 1999 to 2006 and those published from 2007 to 2016), geographical area (comparison between studies conducted in Brazil and elsewhere), study design (whether the data collection was cross-sectional or non-cross-sectional), sample size (continuous variable), and quality score (whether the quality score was ≤10 or >10). We also performed an analysis considering two groups of studies: those published during the period from 1999 to 2006 and those published during the period from 2007 to 2016. In the studies published within the last ten years (2006 to 2016), we also analyzed the results stratified by sex and age group (<40 years and ≥40 years). All analyses were performed using Stata version 13 (Stat Corp LP, TX, USA) with the commands **metan** (for the random-effects meta-analysis) and **metareg** (for the meta-regression).

## Results

**Fig. 1:**
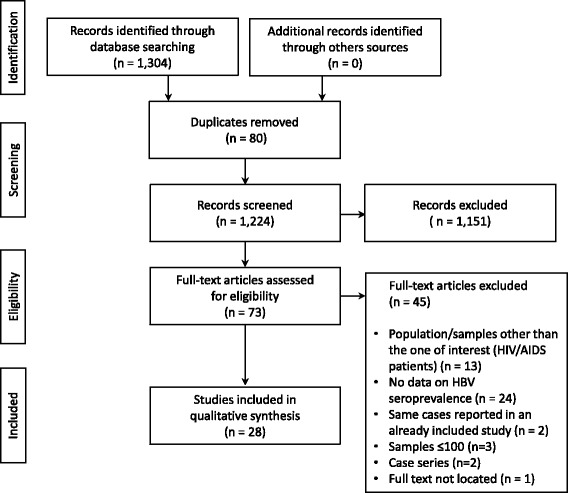
Flowchart of the identification, inclusion, and exclusion of the studies

We identified 1304 publications in Medline, Lilacs, and Embase, and no additional articles were found by the manual search (Fig. 1). After excluding duplicates (80), 1224 articles were selected for reading of the titles and abstracts.

After reading, 1151 articles were excluded, and 73 articles were selected for reading of the full text. Of these, we selected 28 articles (*n* = 18,457) published from 1999 to 2016 for data extraction.

Of the 28 publications on the prevalence of infection with HBV in PLWHA, 18 studies (64.3%) were from Brazil, 3 (10.7%) were from Argentina, 2 (7.1%) were from Chile, 2 (7.1%) were from Cuba, 1 (3.6%) was from Colombia, 1 (3.6%) was from Venezuela, and 1 (3.6%) was from Jamaica (Table 1). The sample sizes ranged from 129 to 2994 participants (mean: 659; median: 471). The mean score of the study quality assessment was 11.6 (range: 8–16). No study was scored ≤7, 25 studies (89.3%) had scores between 8 and 14, and 3 (10.7%) studies had scores between 15 and 21 (maximum score: 21). The most common reasons precluding higher scores were the methods used for sample selection and the presentation of the results (the details of the quality assessment of the studies are shown in the Additional files 2 and 3). The characteristics of the studies and the sample populations are shown in Table 1.

**Table 1:** Prevalence studies of HBsAg in people living with HIV/AIDS

Author	Year	Geographical region	Collection period	Study design	Total	Mean age	Gender (% M)	Outcome	HBsAg + (%)	Quality assessment
Oliveira et al. [23]	2016	Brazil	2009–2010	Cross-sectional	505	37.6	60.2	25	4.9	15
Brandao et al. [24]	2015	Brazil	2011	Cross-sectional	495	40	73.9	19	3.8	14
Vieira et al. [25]	2015	Brazil	2008–2009	Cross-sectional	297	NA	49.8	8	2.6	10
Martins et al. [26]	2014	Brazil	2012–2013	Cross-sectional	300	44.6	59.7	7	2.3	14
Bautista-Amorocho et al. [27]	2014	Colombia	2009–2010	Cross-sectional	275	37.4	65.1	9	3.3	13
Jasper et al. [28]	2014	Venezuela	2002–2011	Retrospective	418	NA	64.1	13	3.1	10
Freitas et al. [29]	2014	Brazil	2009–2011	Cross-sectional	848	41.6	57	21	2.5	13
Oliveira et al. [30]	2014	Brazil	2006–2008	Cross-sectional	768	NA	65.4	46	6	11
Otto-Knapp et al. [31]	2013	Chile	2001–2007	Retrospective	1907	37.2	84.8	161	8.5	12
Tornatore et al. [32]	2012	Brazil	2006–2008	Cross-sectional	130	26.2	0	3	2.3	10
Benzaken et al. [33]	2012	Brazil	2009	Cross-sectional	598	29	47.7	35	5.9	16
Laufer et al. [34]	2010	Argentina	2004–2005	Cross-sectional	593	38	65.6	20	3.3	11
Perez et al. [35]	2009	Chile	1990–2007	Retrospective	311	40.9	90.9	19	6.1	9
Sampaio et al. [36]	2009	Brazil	2004	Cross-sectional	429	39.3	60.1	44	10.3	13
Zago et al. [37]	2007	Brazil	1993–2004	Retrospective	851	35	52.1	32	3.8	13
Quarleri et al. [38]	2007	Argentina	2004–2005	Cross-sectional	593	39	66	22	3.7	11
Braga et al. [39]	2006	Brazil	1998–2003	Retrospective	704	NA	65.1	45	6.4	12
Tovo et al. [40]	2006	Brazil	NA	Retrospective	343	34.4	62.4	14	4.6	10
Grinsztejn et al. [41]	2006	Brazil	1996–2004	Cross-sectional	458	35.4	0	8	1.9	9
Pereira et al. [42]	2006	Brazil	2004	Cross-sectional	1000	37.2	53	37	3.7	13
Corredor et al. [43]	2005	Cuba	2000–2004	Retrospective	2994	NA	NA	309	10.3	8
Monteiro et al. [44]	2004	Brazil	1999–2004	Cross-sectional	406	34.2	NA	32	7.9	15
Souza et al. [45]	2004	Brazil	1992–1995	Retrospective	401	NA	64.8	34	8.5	13
Pavan et al. [46]	2003	Brazil	1992–1995	Retrospective	232	30.8	69.4	12	5.3	10
Smikle et al. [47]	2003	Jamaica	2001–2002	Retrospective	129	NA	38	19	15	10
Mendes-Correa et al. [48]	2000	Brazil	1996	Retrospective	1693	NA	68.2	96	5.7	10
Rodriguez et al. [49]	2000	Cuba	NA	Cross-sectional	295	30	72.5	15	5.1	10
Fainboim et al. [50]	1999	Argentina	1994–1995	Cross-sectional	484	29	74.2	70	14.5	10

**Fig. 2:**
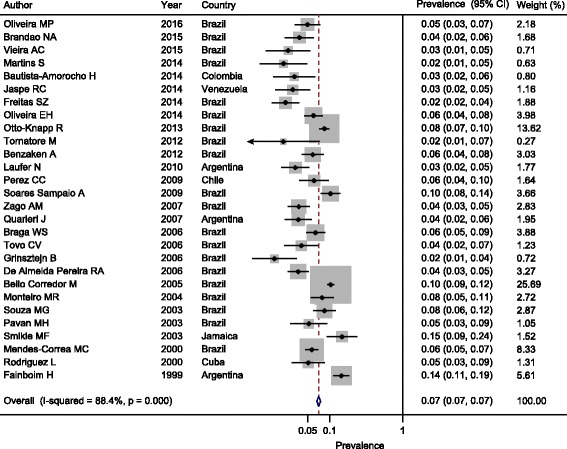
Estimated pooled prevalence of HBsAg in the LAC region

The estimated prevalence of HBsAg in the 28 selected studies in LAC ranged from 2.0% (95% CI 1.0–5.0%) to 15.0% (95% CI 9.0–24.0%); the pooled prevalence was 7.0% (95% CI 7.0–7.0%). The heterogeneity found was substantial among the estimates (I2 = 88.4%, *p* = 0.00) (Fig. 2).

A possible source of heterogeneity may have been the period in which the selected studies were published (from 1999 to 2006 or 2007–2016) (meta-regression coefficient: −0.410846; *p* = 0.033) (Table 2). None of the other factors investigated was significantly associated with heterogeneity.

**Table 2:** Univariate meta-regression analysis of the prevalence of HBV infection among individuals living with HIV/AIDS

	Meta-regression coefficient	95% CI	P
Publication year (1999–2006 vs. 2007–2016)	−0.410846	−0.789861 to −0.032406	0.033
Country (Brazil vs. other)	−0.3252341	−0.7287418 to 0.0782735	0.114
Data collection (cross-sectional vs. other)	−0.3525707	−0.7476732 to 0.0425319	0.080
Sample size	−0.0002151	−0.0000918 to 0.000522	0.170
Quality score	−0.0212113	−0.1197399 to 0.0773112	0.673

**Fig. 3:**
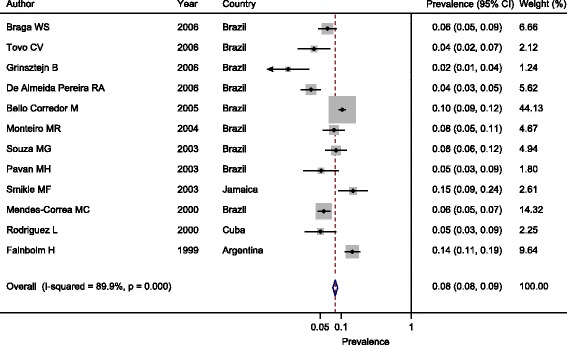
Estimated pooled prevalence of HBsAg in the 12 studies published during the period from 1999 to 2006

**Fig. 4:**
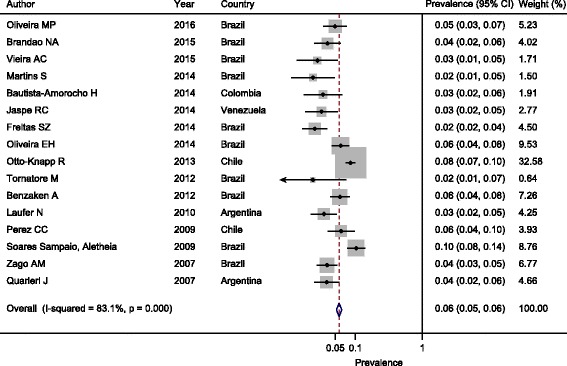
Estimated pooled prevalence of HBsAg in the 16 studies published during the period from 1999 to 2006

Figure 3 shows that the estimated pooled prevalence of HBsAg was 8.0% (95% CI 8.0–9.0%) in the 12 studies published from 1999 to 2006 and 6.0% (95% CI 5.0–6.0%) in the remaining selected studies published from 2007 to 2016 (Fig. 4). Considering the second study period (2007 to 2016), the estimated prevalence of HBsAg for males in seven selected studies ranged from 3.0% (95% CI 1.0–8.0%) to 10.0% (95% CI 8–11%); the pooled prevalence was 8.0% (95% CI 7.0–9.0%) (Additional file 4). For females (8 studies), the estimated prevalence of HBsAg ranged from 1.0% (95% CI 0.0–5.0%) to 4.0% (95% CI 3.0–8.0%); the pooled prevalence was 2.0% (95% CI 2.0–3.0%) (Additional file 5). For the participants aged ≥40 years (3 studies), the pooled prevalence was 5.0% (95% CI 4.0–6.0%) (Additional file 6), whereas in participants younger than 40, the pooled prevalence was 3.0% (95% CI 2.0–4.0%) (Additional file 7).

During the period from 1999 to 2006, 3 articles were published that analyzed the factors associated with HBV exposure. The factors most frequently associated with HBV infection were increasing age and homosexuality. During the later period (2007 to 2016), six studies performed the same analysis. The factors most frequently associated with HBV were the male sex, increasing age, intravenous drug use, and a history of sexually transmitted diseases.

## Discussion

HBV infection is one of the leading causes of morbidity and mortality among people living with HIV/AIDS and other populations with immune deficiencies. In our review of the results of the available studies, we attempted to present a comprehensive overview of the literature on the subject and gain new insights into the distribution of HBsAg among PLWHA.

We systematically reviewed the studies conducted in LAC and found 28 studies from 7 countries (*n* = 18,457). Our review indicated that the pooled prevalence of hepatitis B in PLWHA in LAC was approximately 7.0%. In studies published during the last 10 years, the estimated pooled prevalence of HBsAg among PLWHA was 6.0%. During the latter period, the prevalence of HBsAg in male participants and participants aged ≥40 years was higher than the prevalence in women and in participants younger than 40 years of age.

We did not find any review study on the prevalence of hepatitis B among PLWHA in LAC. In areas of low endemicity, such as the United States and Europe, HBV and HIV are usually acquired in adulthood by sexual and percutaneous transmission [17]. In these regions, the prevalence of HBV coinfection among individuals with HIV varies from 5% to 10% depending on the route of infection. Konopnicki et al. [2] reported data on the prevalence of HBsAg in the EuroSIDA study. This prospective observational cohort study evaluated patients with HIV-1 from 72 centers located in Europe and, more recently, in Argentina and Israel. The authors found that 498 of 5728 patients (8.7%) were positive for HBsAg. The highest prevalence rates for HBsAg were found in Argentina (17.8%), northern and central Europe (9.1%), southern Europe (8.9%), and eastern Europe (5.9%). This result suggests that the prevalence of chronic hepatitis B in this group is 100-fold higher than the prevalence in the general population [2]. A study of patients with HIV infection from 11 geographic regions in the United States [11] indicated that the HBsAg prevalence was 7.6%, and it was higher in male homosexuals than in heterosexuals; moreover, the prevalence of HBV was 2.3% in participants who used antiretroviral drugs containing lamivudine and 7.8% among participants who did not use lamivudine. In countries with intermediate and high endemicity, the main transmission routes were perinatal and during childhood. Generally, HBV infection precedes HIV infection by decades [17]. In the endemic area of Sub-Saharan Africa, HBsAg is found in up to 36% of the HIV-infected population [18]. The authors of one systematic review investigated the prevalence of hepatitis B and C in Sub-Saharan Africa and found rates ranging from 1.1% to 35.7%. The highest rates ​​were found in western African regions (Nigeria, Ghana, Ivory Coast, Gambia, and Burkina Faso). In a study conducted in patients from the TREAT Asia HIV Observational Database (TAHOD), which involved a multicenter cohort of patients with HIV in the Asia-Pacific region, the authors reported that 10.4% (591/5656) of the participants were HBsAg-positive [19]. A study conducted in China evaluated the prevalence of hepatitis B in 1958 HIV-infected patients [20]. Of the 1958 patients, 186 (9.5%) were HBsAg-positive. The rates varied widely depending on the studied region of the country; the participants from the eastern region had the highest prevalence (14.5%), whereas those from the central region had the lowest prevalence (5.0%).

Both tropical and central Latin America exhibited a significant decrease in the prevalence of HBsAg between 1990 and 2005 [21]. Tropical Latin America (Brazil and Paraguay) was reclassified from an intermediate endemicity region to a low endemicity region. Similarly, the prevalence in central Latin America (Colombia, Costa Rica, El Salvador, Guatemala, Honduras, Mexico, Nicaragua, Panama, and Venezuela) decreased during this period, and a shift to low endemicity was observed for most age groups in 2005. In other regions, such as Andean Latin America (Bolivia, Ecuador, and Peru) and South Latin America (Argentina, Chile, and Uruguay), the endemicity levels remained intermediate even though the prevalence decreased with age.

The heterogeneity of the data on the prevalence of HBsAg in PLWHA also significantly limited the interpretation of the results. However, the analysis shown in Table 3 (prevalence of HBsAg in the general population in selected countries based on data by Schweitzer et al. [22]) indicated that the actual prevalence of HBsAg in PLWHA was highly likely to be greater than the prevalence in the general population. The reported prevalence in studies published during the last ten years (Fig. 4) suggests that the prevalence of HBsAg among PLWHA is on average 6.7 times (range: 1.5 to 15.9) higher than that in the general population. Additionally, the overall prevalence of HBsAg in Brazil, Colombia, and Venezuela decreased when comparing the data from 1990 and 2005 [21]. The countries shifted from an intermediate endemicity region (2–8%) to a low endemicity region (<2%), likely due to the implementation of vaccination against hepatitis B in children younger than 1 year of age in 1998. However, the prevalence of HBsAg in PLWHA appeared to remain between 2% and 8%.

**Table 3:** Prevalence of HBsAg in the general population in the selected countries according to Schweitzer et al. [22]

	Prevalence of HBsAg	
Country	%	95% CI
Argentina	0.77%	0.77–0.78
Brazil	0.65%	0.65–0.66
Chile	0.68%	0.34–1.35
Colombia	2.29%	1.86–2.82
Cuba	1.30%	0.62–2.70
Jamaica	3.76%	2.65–5.29
Venezuela	0.48%	0.44–0.52

The available data from the last 10 years showed that male individuals aged ≥40 years were the most affected by HBV infection. Age is a known risk factor associated with HBV, likely due to increasing exposure over time. We observed that the male sex was also a risk factor, likely due to the more frequent exposure to risk factors involved in HBV transmission, especially sexual contact.

The results of this review indicate that the prevention of HBV infection in PLWHA and HIV infection in the population with chronic hepatitis B has not been effective in LAC. Of the known strategies to prevent HBV infection, the most efficient is universal vaccination. In addition to immunization against hepatitis B, active recognition of chronic hepatitis B carriers is necessary. This measure is necessary to allow this population to receive specific treatment and to prevent the progression to well-known complications, which are more severe and rapidly progressive in patients with immunodeficiency, and the spread of infection, which is transmitted primarily via the sexual and vertical routes.

This study has limitations. Studies on the prevalence of HBsAg are unavailable in various regions. Population-based studies are rare in all LAC regions, including areas in which the prevalence of HBsAg in the general population is at the intermediate level of endemicity, such as Belize (4.71%), the Dominican Republic (4.09%), Jamaica (3.76%), and Suriname (3.9%), and those with high levels of endemicity, such as Haiti (13.55%) [22]. Thus, more studies are necessary to elucidate ​​the burden of coinfection with HIV and HBV in LAC. However, our results indicate the need to increase the investment in preventive measures against hepatitis B, particularly when the impact of adequate vaccination in this population is considered.

## Conclusions

The estimated pooled prevalence of HBsAg among PLWHA in the selected studies was 7.0% (95% CI 7.0–7.0%), and the prevalence was 6.0% in the studies published within the last 10 years. The heterogeneity found was substantial among the estimates (I2 = 88.4%, *p* = 0.00). A possible source of heterogeneity was the period in which the selected studies were published (from 1999 to 2006 or 2007–2016) (meta-regression coefficient: −0.410846; *p* = 0.033).

## Additional files

Additional file 1: Prisma 2009 Checklist. (PDF 85 kb)

Additional file 2: Instrument for the assessment of the quality of the studies. Describes the items considered for the assessment of the quality of the studies. (PDF 64 kb)

Additional file 3: Assessment of the quality of the studies. Contains data corresponding to the quality of the studies that describe their (quality) total score relative to the following items: sampling process, procedures used for data collection, and data analysis and description. (PDF 68 kb)

Additional file 4: Estimated pooled prevalence of HBsAg in males during the period from 2007 to 2016 in Latin America and the Caribbean. (PDF 276 kb)

Additional file 5: Estimated pooled prevalence of HBsAg in women during the period from 2007 to 2016 in Latin America and the Caribbean. (PDF 362 kb)

Additional file 6: Estimated pooled prevalence of HBsAg in individuals aged 40 years and over during the period from 2007 to 2016 in Latin America and the Caribbean. (PDF 309 kb)

Additional file 7: Estimated pooled prevalence of HBsAg in individuals under 40 years of age during the period from 2007 to 2016 in Latin America and the Caribbean. (PDF 259 kb)

## Abbreviations

AIDS: Acquired immune deficiency syndrome
HBV: Hepatitis B virus
HIV: Human immunodeficiency virus
LAC: Latin America and the Caribbean
PLHA: People living with HIV/acquired immune deficiency syndrome
