# Supplementary material for: Prevalence of hepatitis B in people living with HIV/AIDS in Latin America and the Caribbean: a systematic review and meta-analysis
Source: BMC Infect Dis. 2017 Aug 24;17:587. doi: 10.1186/s12879-017-2695-z (PMC5571507; doi:10.1186/s12879-017-2695-z)
Supplement: Supplementary file 2 — Instrument for the assessment of the quality of the studies. Describes the items considered for the assessment of the quality of the studies. (PDF 64 kb) [file 12879_2017_2695_MOESM2_ESM.pdf]

Additional file 2. Instrument for assessment of the quality of the studies

Title: ....
Authors: ....
Yes: ....

Answer yes (Y) or no (N):

1. Is the study design adequate for its aims?...|__|
2. Were the data prospectively collected? ....|__|
3. Was the target population clearly defined?...|__|
4. Was probabilistic sampling used to identify potential participants? ....|__|
5. Was the sample size calculated? ....|__|
6. Were the inclusion and exclusion criteria well defined? ....|__|
7. Was the study period specified? ....|__|
8. Was the age range specified? ....|__|
9. Was the selection of participants adequate? (Did the authors comply with the preset criteria?) ......|__|
10. Is the proportion of non-participants adequate? Less than 30%? ....|__|
11. Is the sample of participants representative of the target population? ....|__|
12. Was the data collection standardized? ....|__|
13. Was the outcome clearly defined?..|__|
14. Is the outcome measure adequately described? (detection method?)..|__|
15. Is the method used for outcome diagnosis valid?...|__|
16. Was the data analysis described in a complete manner? ....|__|
17. Was the total number of participants clearly reported? ....|__|
18. Was the number of individuals who experienced the outcome clearly reported? ....|__|
19. Were the prevalence rates also described by age and sex? ....|__|
20. Did the authors report the 95% CI of the prevalence rates? ....|__|
21. Are the reported CI satisfactory? ....|__|

Total score |____|
