# Supplementary material for: Prevalence of hepatitis B in people living with HIV/AIDS in Latin America and the Caribbean: a systematic review and meta-analysis
Source: BMC Infect Dis. 2017 Aug 24;17:587. doi: 10.1186/s12879-017-2695-z (PMC5571507; doi:10.1186/s12879-017-2695-z)
Supplement: Supplementary file 3 — Assessment of the quality of the studies. Contains data corresponding to the quality of the studies that describe their (quality) total score relative to the following items: sampling process, procedures used for data collection, and data analysis and description. (PDF 68 kb) [file 12879_2017_2695_MOESM3_ESM.pdf]

Additional file 3. Assessment of the quality of the studies

AUTHOR	SAMPLE	COLLECTION	PRESENTATION	TOTAL SCORE
MARTINS S	7	4	3	14
BAUTISTA-AMOROCHO H	5	3	5	13
JASPE RC	3	4	5	12
FREITAS SZ	4	4	5	13
OLIVEIRA EH	2	4	5	11
OTTO-KNAPP R	7	2	3	12
TORNATORE M	6	2	2	10
BENZAKEN A	6	4	6	16
LAUFER N	5	2	4	11
PEREZ CC	4	3	1	8
BELLO CORREDOR M	3	3	2	8
ZAGO AM	5	4	4	13
QUARLERI J	5	4	2	11
BRAGA WS	6	4	2	12
TOVO CV	4	4	2	10
GRINSZTEJN B	4	2	3	9
DE ALMEIDA PEREIRA RA	6	4	3	13
MONTEIRO MR	7	4	5	16
SOUZA MG	5	4	4	13
PAVAN MH	3	4	3	10
SMIKLE MF	3	4	3	10
FAINBOIM H	4	4	2	10
MENDES-CORREA MC	4	4	2	10
RODRIGUEZ L	3	4	3	10
SOARES-SAMPAIO A	6	4	3	13
BRANDAO NA	5	4	5	14

VIEIRA AC	5	4	2	11
OLIVEIRA MP	7	4	5	16
