# Supplementary figures and images for: Prevalence of hepatitis B in people living with HIV/AIDS in Latin America and the Caribbean: a systematic review and meta-analysis
Source: BMC Infect Dis. 2017 Aug 24;17:587. doi: 10.1186/s12879-017-2695-z (PMC5571507; doi:10.1186/s12879-017-2695-z)

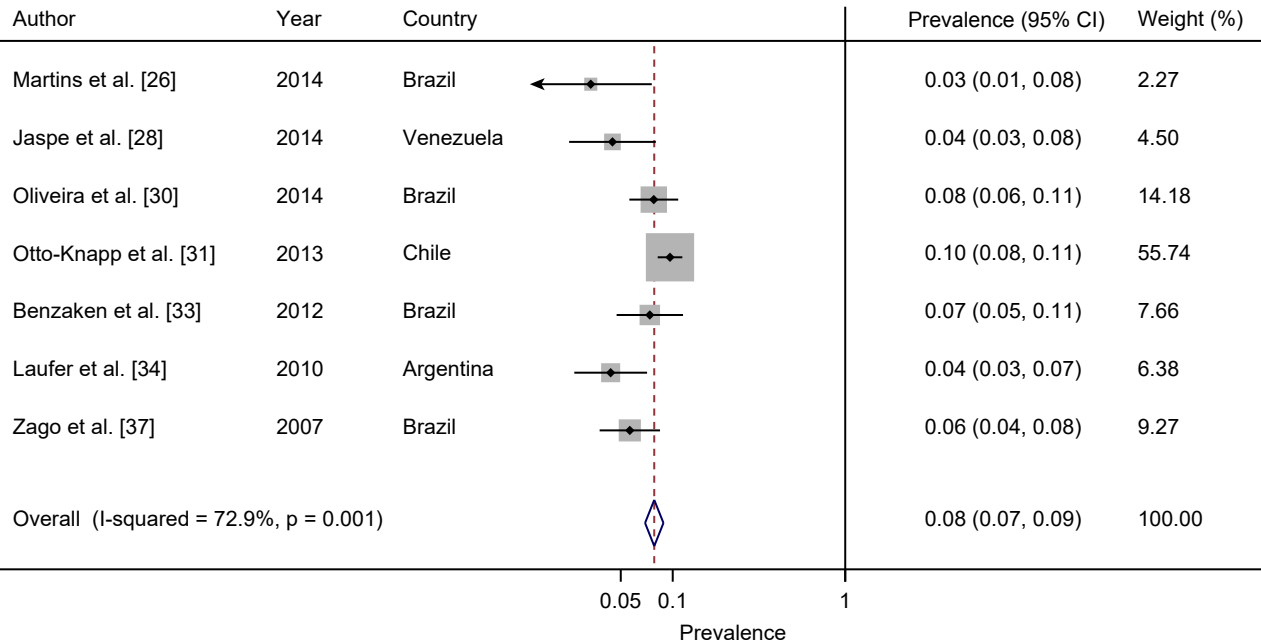

Supplement: Supplementary file 4 — Estimated pooled prevalence of HBsAg in males during the period from 2007 to 2016 in Latin America and the Caribbean. (PDF 276 kb) [file 12879_2017_2695_MOESM4_ESM.pdf]

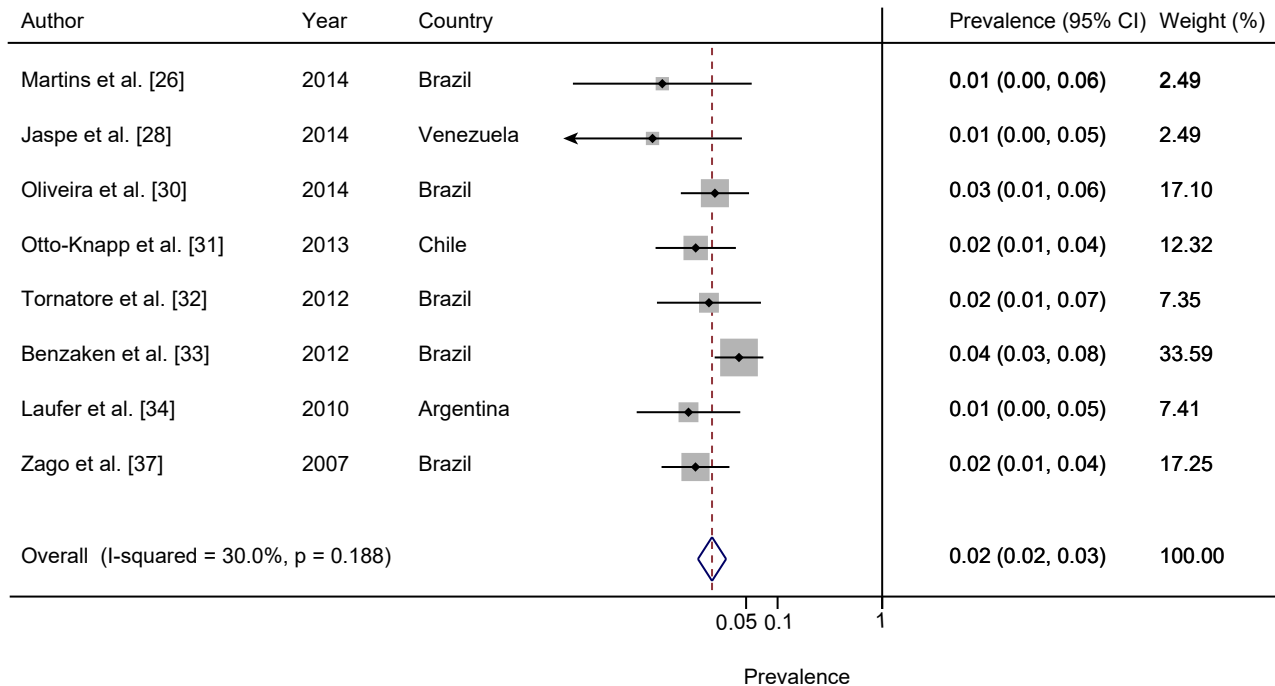

Supplement: Supplementary file 5 — Estimated pooled prevalence of HBsAg in women during the period from 2007 to 2016 in Latin America and the Caribbean. (PDF 362 kb) [file 12879_2017_2695_MOESM5_ESM.pdf]

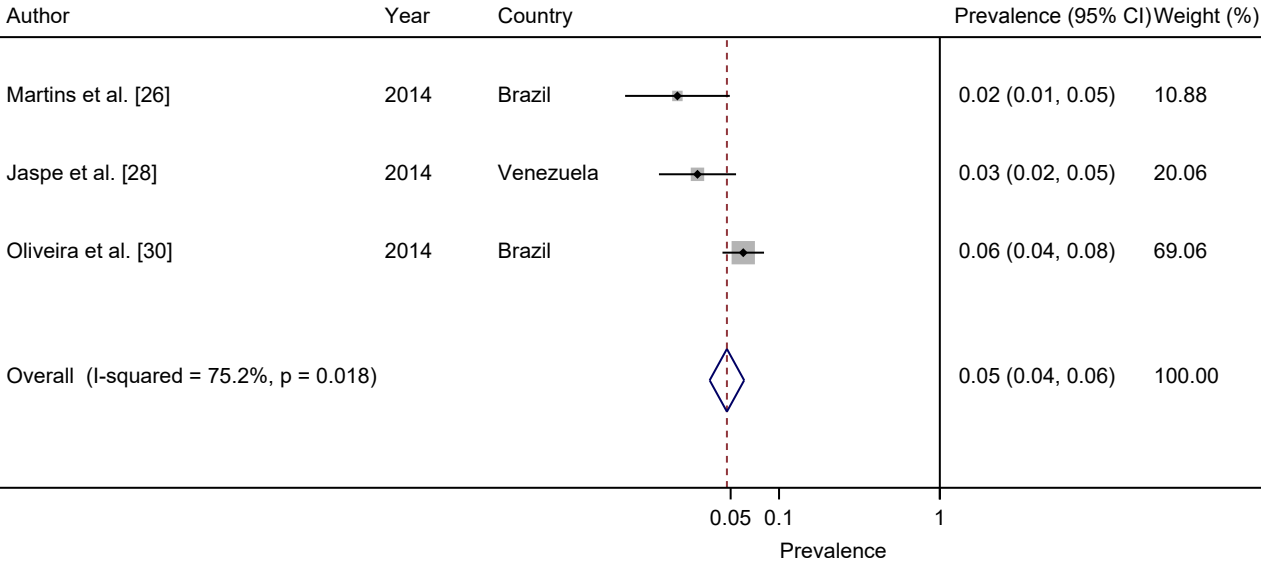

Supplement: Supplementary file 6 — Estimated pooled prevalence of HBsAg in individuals aged 40 years and over during the period from 2007 to 2016 in Latin America and the Caribbean. (PDF 309 kb) [file 12879_2017_2695_MOESM6_ESM.pdf]

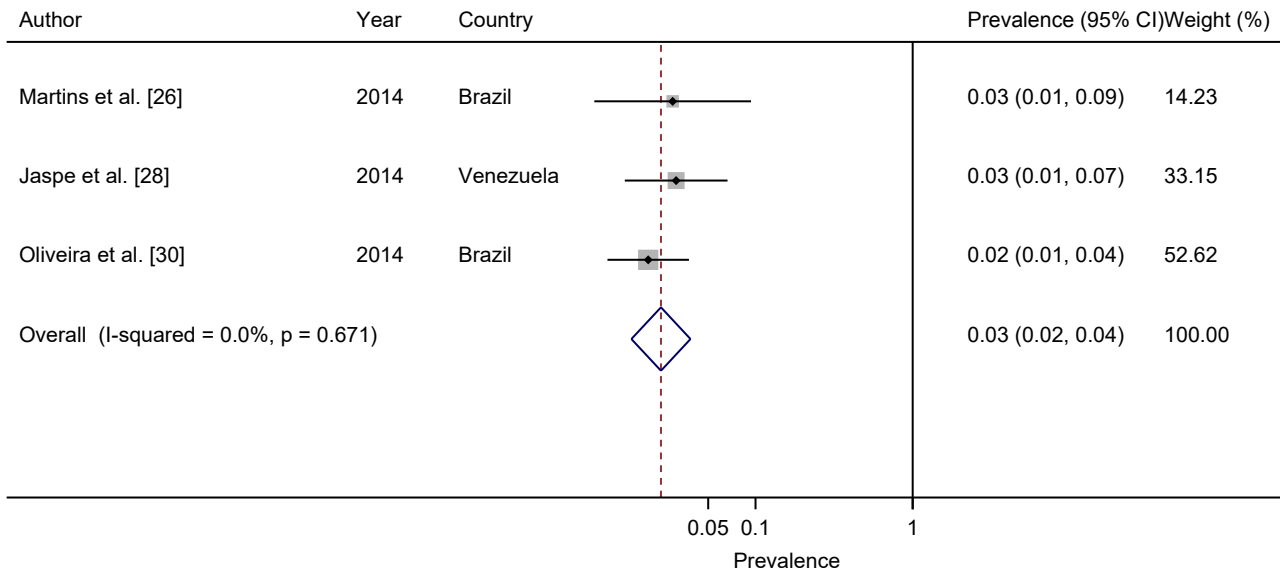

Supplement: Supplementary file 7 — Estimated pooled prevalence of HBsAg in individuals under 40 years of age during the period from 2007 to 2016 in Latin America and the Caribbean. (PDF 259 kb) [file 12879_2017_2695_MOESM7_ESM.pdf]
